# Similar alteration for mental and physical aspects in health-related quality of life over 5 to 8 years in 1347 patients with early arthritis and early inflammatory back pain

**DOI:** 10.1186/s13075-019-1841-y

**Published:** 2019-02-19

**Authors:** Déborah Puyraimond-Zemmour, Benjamin Granger, Anna Molto, Cécile Gaujoux-Viala, Francis Guillemin, Adeline Ruyssen-Witrand, Maxime Dougados, Bruno Fautrel, Laure Gossec

**Affiliations:** 1 Sorbonne Université, Institut Pierre Louis d’Epidémiologie et de Santé Publique, GRC-UPMC 08 (EEMOIS), Paris, France; 2 Department of rheumatology, AP-HP, Pitié Salpêtrière Hospital, Paris, France; 3 Department of Biostatistics, Public Health and Medical Information, AP-HP, Pitié Salpêtrière Hospital, Paris, France; 4 Paris Descartes University, Department of Rheumatology- HopitalCochin, Paris, France; 5 INSERM (U1153): Clinical epidemiology and biostatistics, PRES Sorbonne Paris- Cité, Paris, France; 6 Nîmes University Hospital, Department of Rheumatology, EA 2415, University of Montpellier, 30029 Nîmes, France; 7 Université de Lorraine, EA 4360 APEMAC and Inserm CIC, 1433 Epidémiologie clinique, CHRU de Brabois, Nancy, France; 8 CHU de Toulouse, Hôpital Pierre-Paul Riquet, Toulouse, France; 9 UMR1027, Inserm, Faculté de Médecine, Université Paul Sabatier, Toulouse, France; 10 Hôpital Pitié-Salpêtrière, Service de Rhumatologie, 47-83, boulevard de l’Hôpital, 75013 Paris, France

**Keywords:** Quality of life, Rheumatoid arthritis, Axial spondyloarthritis, Patient outcomes assessment, Early arthritis, Early inflammatory back pain

## Abstract

**Introduction:**

Health-related quality of life (HRQoL) is a priority for patients. The objectives were to describe the changes in HRQoL over 5–8 years in patients with early arthritis (EA) or early inflammatory back pain (IBP) and to explore factors associated to HRQoL.

**Patients and methods:**

In 2 prospective observational French cohorts (ESPOIR for EA patients and DESIR for early IBP patients), HRQoL was assessed regularly over 5–8 years, using the SF36 physical and mental composite scores (PCS and MCS, range 0–100). Disease activity was assessed by DAS28-ESR and ASDAS-CRP. Univariate and multivariate linear mixed-effect models and trajectory-based mapping were applied.

**Results:**

In all, 1347 patients (701 EA and 646 early IBP) were analysed: mean age 48.4 ± 12.2 and 33.9 ± 8.7 years respectively; mean disease duration 3.4 ± 1.7 and 18.2 ± 10.8 months; and 76.3% and 55.0% females. At baseline, in EA, mean PCS and MCS were respectively 40.2 ± 9.1 and 40.4 ± 11.2 and, in early IBP, were respectively 38.5 ± 8.5 and 39.8 ± 10.9. Over follow-up, HRQoL mean levels improved mostly over the first 6 months (*p* <  0.001). Two trajectories were evidenced in both diseases. The ‘good HRQoL’ trajectory groups, i.e. 54–61% of patients, reached levels of HRQoL close to population norms. DAS28-ESR and ASDAS-CRP over time were related to PCS (range of explained variance 9–43%, *p* <  0.001 in the mixed models) but not to MCS.

**Conclusion:**

HRQoL was altered similarly for both physical and mental aspects in EA and early IBP. Disease activity only partly explained HRQoL: the drivers of HRQoL should be further explored.

**Electronic supplementary material:**

The online version of this article (10.1186/s13075-019-1841-y) contains supplementary material, which is available to authorized users.

## Background

Health-related quality of life (HRQoL) is a priority for patients living with a chronic disease [1, 2]. HRQoL is a broad ranging concept incorporating in a complex way the person’s physical health, psychological state, level of independence, and social relationships [1, 3]. HRQoL is recognized as an important outcome both in rheumatoid arthritis (RA) and axial spondyloarthritis (axSpA) and preservation or improvement of HRQoL should be one of the objectives of RA and axSpA management [4–7]. HRQoL is frequently measured with the 36-Item Short Form Survey (SF-36), a generic questionnaire assessing both physical and mental components of HRQoL [8].

Several studies have shown that patients with established RA and axSpA have an impaired quality of life for both physical and mental components [9–13]. Due to the period of the diagnosis and to the potential lack of coping mechanisms in early disease versus established disease, HRQoL may be altered differently in early disease versus in established disease [14]. In early disease, such as early arthritis (EA) and early inflammatory back pain (IBP), HRQoL is also altered for patients entering a randomized clinical trial [15, 16]. However, to our knowledge, little information in an observational context is available about HRQoL in early diseases [17].

Furthermore, little is known about the natural history and evolution of HRQoL in these two chronic inflammatory diseases [18]. Data regarding HRQoL in RA and axSpA are either cross-sectional or are issued from selected population included in randomized trials [10, 16, 19–23]. Over a period of several years, HRQoL may be modified either through disease management or through self-management. We hypothesized that HRQoL would be profoundly modified in early disease but would improve over the next years.

HRQoL is very multifactorial and factors contributing to altered HRQoL in established RA and axSpA may include both inflammatory elements (such as inflammatory pain, stiffness and fatigue) and non-inflammatory elements (such as joint destruction or spinal ossification) [22, 24, 25]. Disease activity may be evaluated in RA by the Disease Activity Score 28—erythrocyte sedimentation rate (DAS28-ESR) and in axSpA by the Ankylosing Spondylitis Disease Activity Score—C reactive protein (ASDAS-CRP) [26, 27]. Assessing the link between HRQoL and ASDAS-CRP or DAS28-ESR may give interesting information on these potentially explanatory factors of HRQoL.

Thus, the objectives were to describe the changes in HRQoL over 5 to 8 years in patients with EA and early IBP and to explore factors associated to HRQoL.

## Patients and methods

### Study design

In the present study, two large, prospective, multicenter, longitudinal, observational French cohorts of patients were used. They have been previously described in detail [28, 29]. Briefly, ESPOIR (Etude et Suivi des Polyarthrites Indifférenciées Récentes) included patients with EA and DESIR (Devenir des Spondylarthropathies Indifférenciées Récentes) included patients with early IBP. Here, the first 8 years of ESPOIR and 5 years of DESIR follow-up were analyzed [28, 29].

These studies fulfilled current good clinical practice and obtained the approval of the appropriate ethical committees, and participants gave their written informed consent. Databases locked in February 2017 for DESIR and June 2017 for ESPOIR were used.

### Patients

#### EA patients

A total of 813 patients were included in ESPOIR [25]. These were consecutive patients aged > 18 and < 70 years with EA defined as definite synovitis for less than 6 months and a certain, probable or possible clinical diagnosis of RA.

#### Early IBP patients

A total of 708 patients were included in DESIR [29]. These were consecutive patients aged > 18 and < 50 years with IBP for more than 3 months but less than 3 years and symptoms suggestive of axSpA diagnosis according to the investigator.

At baseline, in EA and early IBP, no patients were taking biological. Conventional DMARDS were prohibited. For our analysis, only patients for whom at least 3 HRQoL assessments were available over follow-up were included.

### Data collection

#### Assessment of HRQoL

HRQoL was assessed with the SF-36, a generic and multi-dimensional questionnaire. SF-36 has 8 subdomains, which can be combined into physical and mental component summary scores, on a scale of 0–100 with higher scores indicating better health status and 50 (standard deviation ± 10) indicated US norms for the general population (French norms of the composite scores for the general population are not available) [29–32]. Population-based norms (for the USA) were also represented for MCS and PCS and equal to 50 (standard deviation 50), and for France for the subdomains [32, 33]. The physical composite score (PCS) includes physical function, physical role, bodily pain and general health and the mental composite score (MCS) includes mental health, emotional role, social function and vitality [30, 31, 34]. SF-36 was assessed at baseline, every 6 months up to month 24, then once a year up to 8 years in EA and up to 5 years in early IBP.

#### Other data collection

Socio-demographic data included age, gender, educational level (above or below end of high school), occupational category (no professional activity/retired/workmen/farmer versus employee/intermediate/high level employment) and smoking status (yes/no) at baseline. For all patients, symptom duration and Health-Assessment Questionnaire (HAQ) were collected [35].

In RA, disease-related data at baseline included rheumatoid factor and anti-CCP antibodies, swollen joint counts and tender joint counts. The percentage of patients fulfilling the ACR/EULAR 2010 criteria classification for RA was calculated [36].

At each visit, use of any DMARDs (yes/no), oral glucocorticoids (yes/no) and disease activity through DAS28-ESR were collected [26]. The presence of erosions on hands and/or feet according to local reading was collected (yes/no) at 4, 6 and 8 years.

In axSpA, disease-related data at baseline included HLAB27 status, radiological or MRI sacroiliitis according to local reading and symptom duration. The ASAS classification criteria were assessed [37]. At each visit, disease activity through ASDAS-CRP and use of Tumor Necrosis Factor inhibitors (TNFi) (yes/no) were collected, as well as extra-articular manifestations (uveitis, psoriasis or inflammatory bowel disease), peripheral articular manifestations and enthesitis. An ASDAS-CRP below 1.3 defines inactive disease and below 2.1, moderate activity [27].

### Statistical analysis

#### Evolution of HRQoL

The mean and standard deviation of SF-36 MCS, SF-36 PCS and the 8 subdomains were calculated at each time-point over 8 years in EA and over 5 years in IBP and represented visually by curves and spidergrams [32]. Population-based norms (for the USA) were also represented for MCS and PCS, and for France for the subdomains [32, 33]. *P* values for trend over time for MCS and PCS scores were calculated by univariate linear mixed-effect models with an ‘intercept’ random effect [38].

To determine multiple homogeneous trajectories rather than a group-level of HRQoL over time, trajectory-based mapping was performed using the k-means design for longitudinal data (klm package in R) [39, 40]. The clusters were checked graphically [40]. Trajectory-based mapping models the relationship of a variable (SF36-MCS and PCS) with time: it defines the shape of the trajectory and the estimated proportion of the population belonging to each trajectory. Each participant is then assigned to the group for which his probability to belong to a trajectory is the highest [39, 40]. All patients included in the mixed models were analysed, and only patients with all HRQoL (MCS and PCS) assessments contributed to the trajectory-based mapping.

Finally, characteristics of patients in each trajectory were described and compared by Student’s *t* and chi-square tests for EA and early IBP.

#### Factors associated to HRQoL

Factors associated to MCS and PCS over 8 years in EA and over 5 years in early IBP were assessed by univariate and multivariate linear random intercept mixed-effect models. Baseline potentially explanatory variables entered in the univariate mixed-effect model were for both disease groups: age, gender, symptom duration, educational level, occupational category, smoking status and baseline SF-36 MCS and PCS. In EA, covariates changing over time were DAS28-ESR, radiographic erosions, DMARDs (yes/no) and oral glucocorticoids (yes/no).

In early IBP, additional baseline variables entered were HLAB27 and radiological or MRI sacroiliitis. Covariates changing over time were ASDAS-CRP, TNFi (yes/no) and extra-articular, peripheral and enthesitic manifestations (yes/no).

Covariates were included in the multivariate model if *p* <  0.20 in univariate analysis. The multivariate analyses were adjusted for baseline MCS and PCS. Baseline variables were included in uni- and multivariate analyses because baseline variables are highly explanatory for later outcomes [41, 42]. Sensitivity analyses were performed in the subgroup of patients fulfilling the ASAS and RA classification criteria [36]. To illustrate the contribution of the disease activity in the multivariate mixed models, the explained variance (*R*^2^) was calculated by univariate logistic regression at baseline and final assessments for indicative purposes.

All analyses were performed using 2009–2016 R version 1.0.143 [43].

## Results

Available data concerned 701 patients at baseline and 508 at 8 years in EA and 646 patients at baseline and 435 at 5 years in early IBP. Thus, 1347 patients contributed to the mixed models and 1025 (566 EA and 459 early IBP) contributed to the trajectory-based mapping.

### Patients characteristics

In all, 1347 patients were analysed: of the 813 ESPOIR patients, 701 had at least 3 SF-36 assessment available over 8 years. Of these, 568 (81.4%) satisfied the RA classification criteria. At baseline, mean age was 48.4 ± 12.2 years; 535 (76.3%) were females; mean swollen joint count was 8.4 ± 7.0; mean tender joint count was 7.4 ± 5.4; 229 (32.9%) had rheumatoid factor and/or anti CCP antibodies; mean DAS28-ESR was 5.12 ± 1.30; and 639 (91.2%) were considered in moderate/high disease activity (Table 1) [26]. Over follow-up, 190 (27.10%) received a biologic.

**Table 1:** Baseline characteristics of 701 patients in early arthritis (EA) and 646 patients in early inflammatory back pain (IBP) patients recruited to observational cohorts

Characteristics	EA population *N* = 701	Patients fulfilling RA classification criteria *N* = 568	IBP population *N* = 646	Patients fulfilling ASAS classification criteria *N* = 402
Age (years), mean (SD)	48.4 (12.2)	48.9 (12.0)	33.9 (8.7)	31.7 (7.3)
Symptom duration (month), mean (SD)	3.4 (1.7)	3.3 (1.7)	18.2 (10.8)	18.2 (10.8)
Sex, females, *N* (%)	535 (76.3)	433 (76.2)	355 (55.0)	197 (49.0)
Studies above high school, *N* (%)	230 (32.8)	180 (31.7)	391 (60.7)	259 (64.4)
Work status: intermediate/high level employment, *N* (%)	532 (75.9)	434 (76.4)	494 (77.1)	291 (72.9)
Smoking status, yes, *N* (%)	326 (46.5)	263 (46.3)	225 (35.0)	152 (37.9)
HAQ, mean (SD)	0.98 (0.69)	1.05 (0.69)	0.67 (0.51)	0.57 (0.49)
DAS28-ESR/ASDAS-CRP, mean (SD)	5.12 (1.30)	5.37 (1.23)	2.62 (0.93)	2.61 (0.99)
SF36-PCS, mean (SD)	38.5 (8.5)	37.8 (8.3)	40.2 (9.1)	40.6 (9.0)
SF36-MCS, mean (SD)	39.8 (10.9)	39.5 (10.7)	40.4 (11.2)	41.0(11.4)

DAS28-ESR was applied to EA and ASDAS-CRP was applied to early IBP. All percentages are calculated on available data

*HAQ* Health Assessment Questionnaire Disability Index, *DAS-28-ESR* Disease Activity Score—erythrocyte sedimentation rate, *ASDAS-CRP* Ankylosing Spondylitis Disease Activity Score*—*C reactive protein, *PCS* physical composite score, *MCS* mental composite score

Of the 708 DESIR patients, 646 had at least 3 SF-36 assessments available over 5 years and were analysed. Of these, 402 (62.2%) satisfied the ASAS classification criteria. At baseline, mean age was 33.9 ± 8.7 years; 255 (55.0%) were females; 177 (27.2%) had extra-articular manifestations; 175 (27.2%) had peripheral arthritis; 362 (56.0%) had enthesitis; 228 (35.3%) had radiological or MRI sacroiliitis; and 379 (58.8%) were HLAB27 positive. Mean ASDAS-CRP was 2.63 ± 0.93, and 571 (92.7%) had moderate or high disease activity [24]. Over the first 5 years of follow-up, 167 patients (25.9%) received TNFi (Table 1).

Patients who were not included in this analysis were similar for early arthritis and for axSpA, had more often studied above high school and had a higher ASDAS-CRP and lower SF36-PCS (data not shown).

### Evolution of HRQoL

#### SF-36 PCS and MCS

**Fig. 1:**
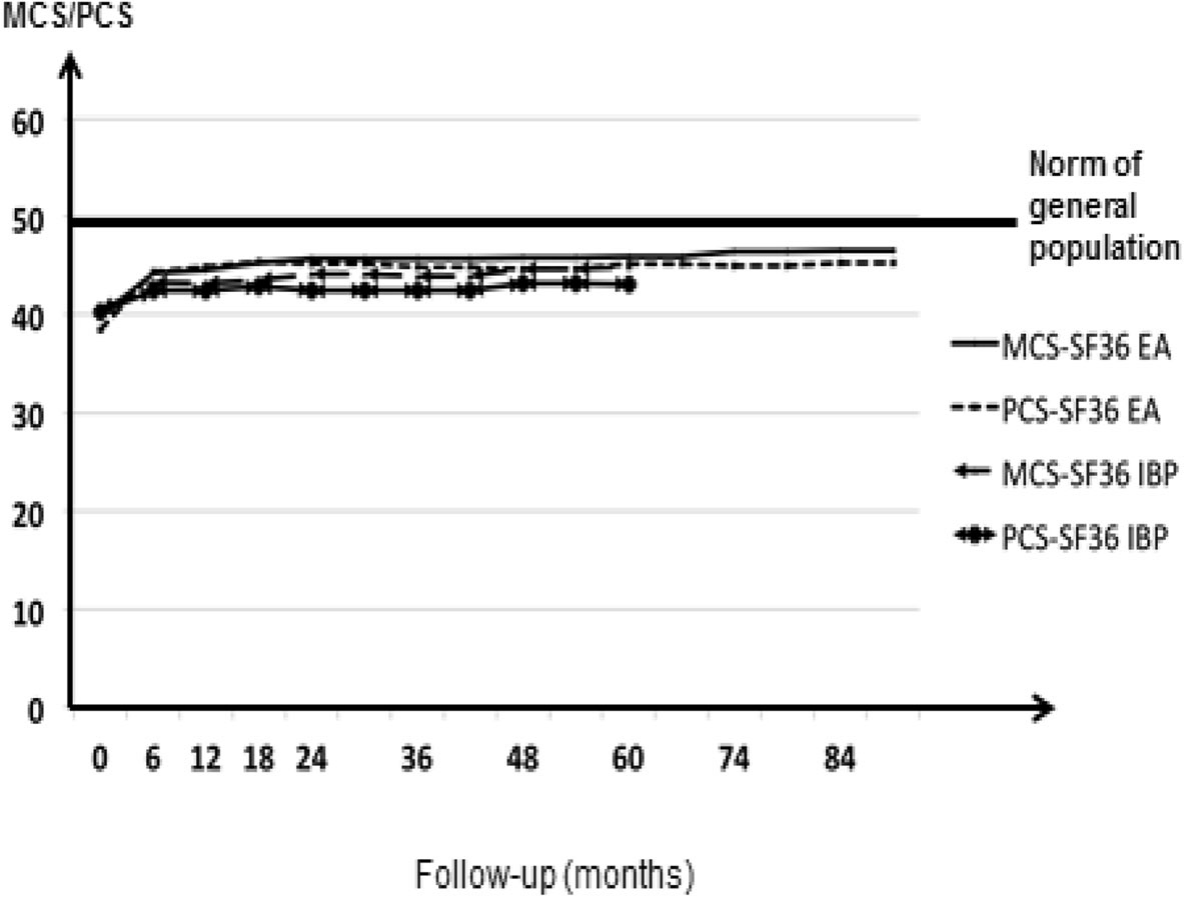
Changes in SF36-MCS and PCS over follow-up in early arthritis (EA) and early inflammatory back pain (IBP). Lines represent mean SF36-MCS and mean SF36-PCS for EA and IBP; 95% confidence intervals were not represented because they were very small. There was no imputation of missing data; thus, the number of available data is between 696 and 508 according to the assessments for SF-36 in EA and 646 to 422 according at the assessment for SF-36 in IBP

At baseline, mean PCS and MCS were respectively 38.5 ± 8.5 and 39.8 ± 10.9 in EA and 40.2 ± 9.1 and 40.4 ± 11.2 in early IBP and were similar for patients fulfilling classification criteria or not (Table 1). At the group level, over follow-up, HRQoL improved significantly (univariate linear mixed-effect models, *p* <  0.001; *t* value for MCS 119 and for PCS 139 in EA and for MCS 115 and PCS 130 in early IBP). The assumption of normal distribution of sampled data for linear models was fulfilled. However, this improvement occurred mostly over the first 6 months (Fig. 1 and Additional file 1: Table S1).

**Fig. 2:**
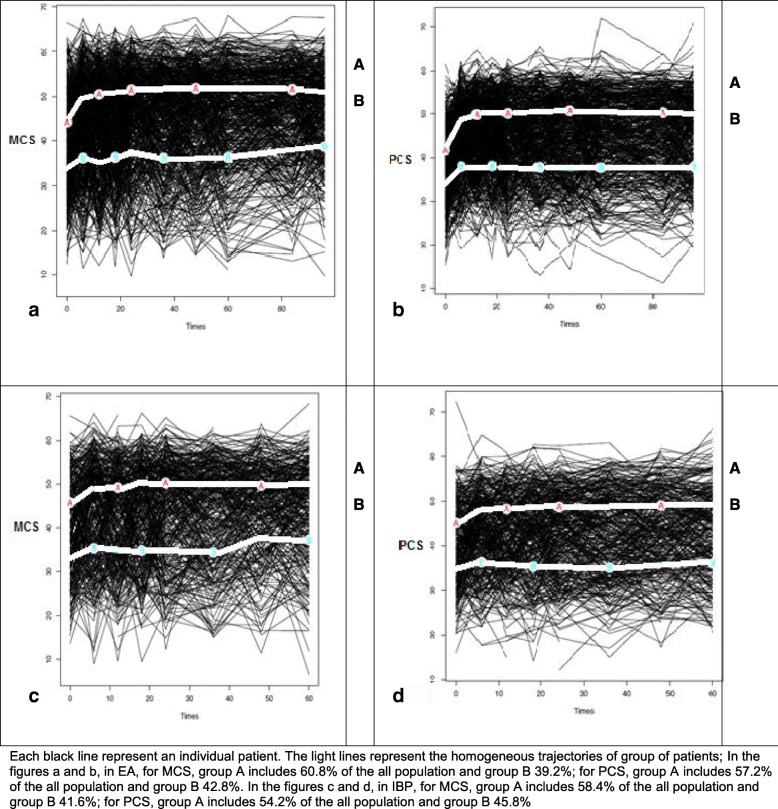
Trajectory-based mapping of HRQoL (MCS and PCS) over 5 to 8 years in patients with early arthritis (EA) (graph **a** and **b**) and early IBP (graph **c** and **d**). Each black line represents an individual patient. The light lines represent the homogeneous trajectories of group of patients. In the figures **a** and **b**, in EA, for MCS, group A includes 60.8% of the all population and group B 39.2%; for PCS, group A includes 57.2% of the all population and group B 42.8%. In the figures **c** and **d**, in IBP, for MCS, group A includes 58.4% of the all population and group B 41.6%; for PCS, group A includes 54.2% of the all population and group B 45.8%

Trajectory-based mapping evidenced two trajectories with an improvement over time but mostly over the first 6 months for each group for both mental and physical scores in both diseases (Fig. 2). Trajectory A or the ‘good HRQoL’ trajectory groups correspond in both diseases, to levels of HRQoL close to those of the general population (SF-36 is a norm-based score with US norms of 50). In all, 57.2–60.8% of EA patients and 54.2–58.4% of early IBP patients were in trajectory A. Trajectory B corresponds to more altered HRQoL (Fig. 2). The 2 trajectories were also evidenced similarly restricting the analysis to patients fulfilling the ACR/EULAR criteria or ASAS classification criteria (data not shown).

Between trajectories, all characteristics at baseline were significantly different (*p* <  0.005), except globally for smoking status and gender with higher complaints and disease activity in the B trajectory **(**Additional file 2: Table S2).

#### Subdomains of SF-36

**Fig. 3:**
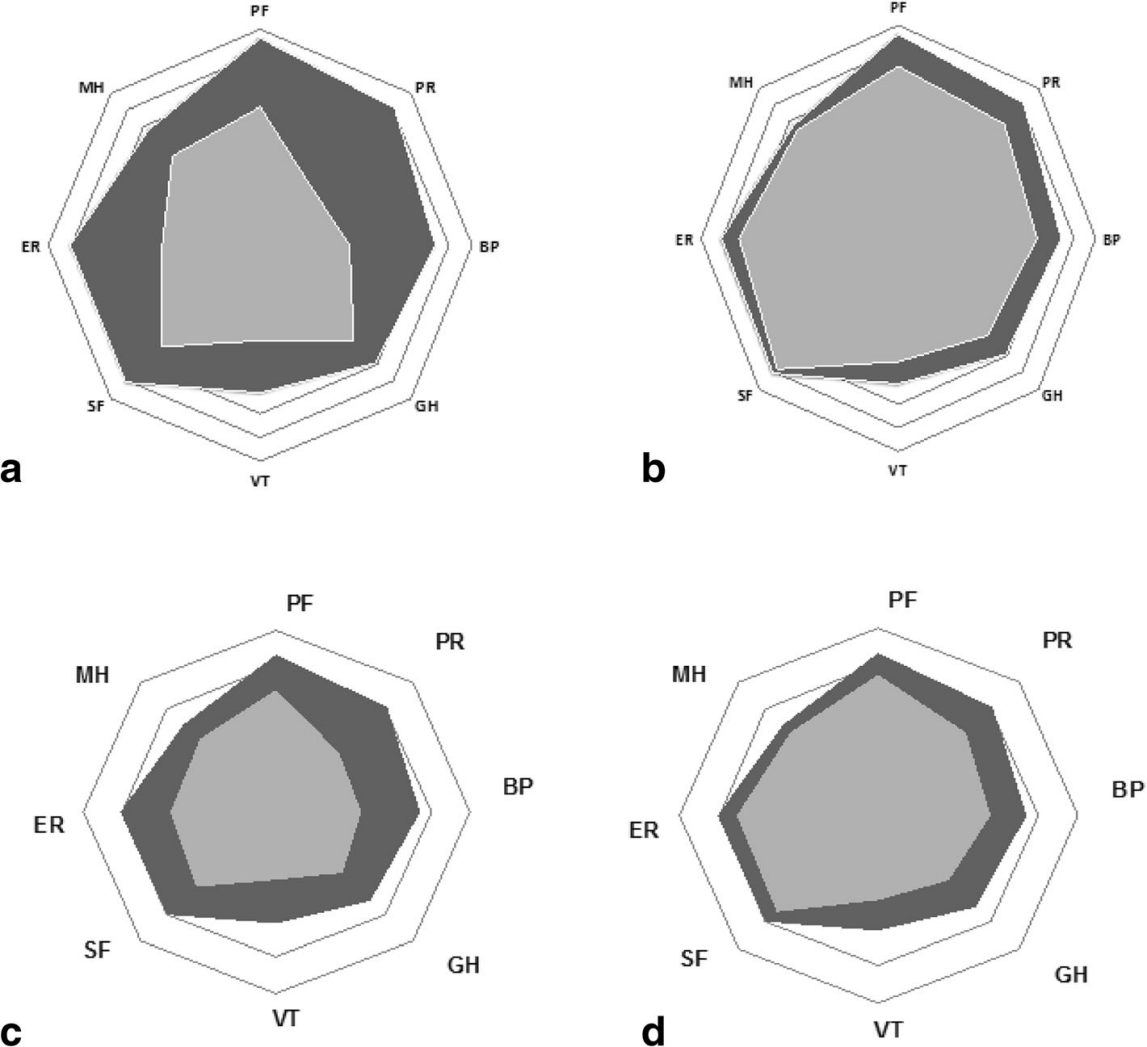
Spidergrams representing the 8 subdomains of SF-36 at baseline and at 5 to 8 years in early arthritis (EA) and early inflammatory back pain (IBP). For each spidergram, domains on the left are included in MCS and on the right in PCS. The outside circle represented the maximum score (100) and the the center of the circle the minimum score (0). The grey portion represents the levels of HRQoL for the early arthritis patients and the dark portion the level of HRQoL for the French norm. Figures **a** and **b**: at baseline (on the left) in 699 patients and at 8 years (on the right) in 510 patients in EA. Figures **c** and **d**: at baseline (on the left) in 642 patients and at 5 years (on the right) in 433 patients in IBP

For both diseases, at baseline, all the subdomains of HRQoL were altered and more in EA (Fig. 3 and Additional file 3: Table S3). Over time, all the SF36 subdomains improved (Fig. 3). This improvement occurred mostly over the first 6 months (Additional file 3: Table S3). Domains with the highest improvement in both patients groups were emotional role and physical role, domains with the lowest improvement were general health and mental health (Fig. 3 and Additional file 2: Table S2).

### Factors related to SF36-MCS and PCS

In EA, in univariate analysis, worse MCS at baseline, higher symptom duration, intake of DMARDs at baseline and higher DAS28-ESR were associated to better MCS over time. In early IBP, in univariate analysis, the variables associated to better MCS were lower baseline MCS and presence of peripheral arthritis. Demographic data and disease activity were associated to PCS in both diseases (Table 2).

**Table 2:** Factors related to SF36-MCS and PCS over 5 to 8 years in early arthritis (EA) and early inflammatory back pain (IBP): results of univariate mixed linear models

Characteristics associated to HRQoL	EA		Early IBP	
	MCS beta (*p* value)	PCS beta (*p* value)	MCS beta (*p* value)	PCS beta (*p* value)
Baseline characteristics				
Age	− 0.0049 (0.055)	− 0.0086 (0.001)	− 0.00031 (0.69)	− 0.0017 (0.003)
Gender	− 0.0056 (0.93)	+ 0.041 (0.50)	+ 0.00075 (0.96)	+ 0.016 (0.12)
Level of studies	− 0.029 (0.66)	+ 0.20 (0.003)	− 0.013 (0.33)	+ 0.017 (0.11)
Level employment	+ 0.017(0.82)	+ 0.12 (0.04)	+ 0.026 (0.06)	− 0.010 (0.32)
Baseline MCS-SF36	− 0.041 (< 0.001)	+ 0.0012 (0.007)	− 0.0083 (< 0.001)	+ 0.0013 (0.004)
Baseline PCS-SF36	− 0.0048 (0.19)	− 0.038 (< 0.001)	− 0.00001 (0.99)	− 0.0052 (< 0.0001)
Smoking status	− 0.097 (0.11)	− 0.049 (0.33)	+ 0.0058 (0.68)	+ 0.0077 (0.46)
HLAB27 status	NA	NA	+ 0.011 (0.42)	− 0.0062 (0.54)
Radiological or MRI sacroiliitis	NA	NA	− 0.012 (0.39)	+ 0.016 (0.12)
Symptom duration	+ 0.0012 (0.04)	+ 0.0025 (< 0.001)	+ 0.0062 (0.42)	− 0.015 (0.008)
Variables changing over time				
Erosions on hands and/or feet	+ 0.54 (0.19)	+ 0.32 (0.36)	NA	NA
Oral corticosteroid	+ 0.13 (0.10)	+ 0.20 (0.002)	NA	NA
DMARDs	+ 0.36 (< 0.001)	+ 0.61 (< 0.001)	NA	NA
DAS28-ESR/ASDAS-CRP	+ 0.20 (< 0.001)	+ 0.27 (< 0.001)	+ 0.0062 (0.39)	− 0.016 (0.0005)
Extra articular manifestations^†^	NA	NA	− 0.0037 (0.80)	+ 0.00001 (1.00)
Peripheral arthritis	NA	NA	+ 0.029 (0.04)	+ 0.018 (0.09)
Enthesitis	NA	NA	+ 0.014 (0.36)	+ 0.015 (0.19)
TNFi	NA	NA	− 0.021 (0.21)	− 0.027 (0.03)

DAS28-CRP was applied to EA and ASDAS-CRP to early IBP

*MCS* mental composite score, *PCS* physical composite score, *ASDAS-CRP* Ankylosing Spondylitis Disease Activity Score—C reactive protein, *DAS28-ESR* Disease Activity Score 28—erythrocyte sedimentation rate, *TNFi* tumor necrosis factor inhibitor, *NA* not analysed

†Extra-articular manifestations including uveitis, psoriasis or inflammatory bowel disease

In multivariate analysis in EA, only worse PCS at baseline was significantly linked to better MCS over time (beta = − 0.049). Lower age (beta = − 0.0064) and lower PCS score at baseline (beta = − 0.041) were significantly linked to better PCS over time. Higher DAS28-ESR, longer studies, baseline DMARDs, higher symptom duration and better MCS score at baseline were linked to better PCS over time (Table 3).

**Table 3:** Factors related to SF36-MCS and PCS over 5 to 8 years in early arthritis (EA) and early inflammatory back pain (IBP): results of multivariate mixed linear models, after adjustment on baseline HRQoL

Factors associated with worst HRQoL	MCS beta (*p* value)	PCS beta (*p* value)
EA		
Age	NS	− 0.0064 (0.010)
DAS28-ESR	NS	+ 0.18 (< 0.001)
Levels of studies	NA	+ 0.26 (0.0001)
DMARDs	NS	+ 0.42 (< 0.001)
Symptom duration	NS	+ 0.0011 (0.048)
Score MCS at baseline	NS	+ 0.013 (< 0.001)
Score PCS at baseline	−0.049 (0.022)	− 0.041 (< 0.001)
Early IBP		
ASDAS-CRP	NA	− 0.028 (< 0.001)
Age	NA	− 0.0012 (0.024)
Peripheral arthritis	NS	− 0.025 (0.0078)
TNFi	NA	− 0.028 (0.013)
Score MCS at baseline	−0.0085 (< 0.0001)	+ 0.0018 (< 0.001)
Score PCS at baseline	+ 0.0018 (0.02)	− 0.0055 (< 0.001)

In EA, oral corticosteroid, smoking status and erosions were analyzed for MCS but were not significant. Oral corticosteroid was analysed for PCS but were not significant. In early IBP, female gender, lower symptom duration, studies above high school, presence of enthesitis, no radiological or MRI sacroiliitis were analysed for PCS but were not significant. Intermediate or high level employment were not analysed for PCS and were not significant for MCS. Both analyses were adjusted on baseline HRQoL

*DAS28-ESR* Disease Activity Score—erythrocyte sedimentation rate, *ASDAS-CRP* Ankylosing Spondylitis Disease Activity Score—C reactive protein, *MCS* mental composite score, *PCS* physical composite score, *TNFi* tumor necrosis factor inhibitor, *NA* not analysed (*p* value > 0.20 in univariate analysis), *NS* not significant

In multivariate analysis, in IBP, apart from baseline HRQoL, no other variable was associated to MCS. Baseline lower ASDAS-CRP, lower age, the absence of peripheral arthritis, non-use of TNFi treatment and better baseline MCS and lower baseline PCS were strongly linked to better PCS over time (Table 3).

For the subgroup of patients fulfilling the ASAS and EULAR/ACR classification criteria, in multivariate analysis, results were globally similar (data not shown).

The variance of PCS explained by ASDAS-CRP and DAS28-ESR was respectively at baseline 27.4% and 34.5% and at last follow-up 9.1% and 43.1% (overall range, 9–43%; 9–47% in early IBP and 34–43% in EA). The variance of MCS explained by ASDAS-CRP and DAS28-ESR was respectively at baseline 14.6% and 6.4% and at last follow-up 17.3% and 0.5% (overall range, 14.6%–17.3% in early IBP and 0.5%–6.4% in EA).

## Discussion

This study brings to light important information on HRQoL in EA and early IBP. At disease inception, HRQoL was altered similarly both for mental and physical aspects. Over a period of 5 to 8 years, HRQoL improved mostly over the first 6 months of follow-up then remained mostly unchanged. In EA and in early IBP, 54–61% of patients reached levels of HRQoL close to population norms. Finally, disease activity only partly explained the physical component of HRQoL, with a stronger link in EA.

In these two populations, HRQoL was altered similarly for physical and mental aspects**.** Compared with the US general population for whom mean levels of PCS and MCS are at 50, mean MCS and PCS were lower in this population of early IBP and EA, as expected. However, 54–61% of patients (trajectories A) reached levels of HRQoL close to population norms for both MCS and PCS-SF36 and both diseases. The patients in trajectories B were worst physically and mentally. The patients in B trajectories in comparison to the A trajectories were mainly of older age and had lower education level and higher complaints and disease activity in both diseases, mentally and physically (Additional file 2: Table S2). Explanations could include the importance of personality traits as well as disease characteristics.

Comparisons with RA and axSpA studies are difficult. In published observational studies of established RA and axSpA, mean PCS was around 32 and 28–56 respectively, and mean MCS was around 39 and 40–66 respectively [9, 11, 44–47] Randomized studies usually include different populations [23]. In clinical trials, although MCS is globally similar, PCS is usually worse [15, 48–51]. Thus, globally, HRQoL appears better for physical aspects and similar or worse for mental aspects in the present study of early disease compared with established disease. Our interpretation is that among EA and early IBP patients, there is a wide diversity in HRQoL levels. The alteration of HRQoL in both diseases observed here appeared more important for MCS and similar for PCS to that observed in cancer in a 2003 US study [52]. All the subdomains of HRQoL were altered in the present study for both EA and early IBP. Compared with French population subdomain norms, EA patients had the results 12.8 to 48.5 points worse and early IBP patients had the results 20 to 35 points worse except for mental health [33]. In both diseases, the most substantial modifications were seen in social interactions (physical role, emotional role) and in pain. Pain was an expected outcome. Perhaps the young age of the patients explains the high perceived impact on social roles: this is a period of life when both professional and family life demands are consequent.

HRQoL was significantly but only slightly improved over the first 5 to 8 years at the group level. This result was confirmed at the individual level by a robust model (trajectory-based mapping). Trajectory analyses identified two clusters; it should be noted that k-means trajectory statistics will always identify at least two groups; thus, the interesting results here are that the method did not identify three or more groups [39, 40]. The initial improvement over the first 6 months may reflect the efficacy of disease management. It may also reflect the efficacy of coping mechanisms [14]. Our hypothesis was that HRQoL would improve over the first few years of disease. The improvement seen was only slight. Physical HRQoL may not be too altered in early axSpA and RA because of the lack of structural damage [53]. On the other hand, MCS was more altered perhaps due to the stress of the diagnosis period. Baseline levels of HRQoL were only moderately altered, perhaps because coping mechanisms were in place already at inclusion in DESIR, which happened 18 months after the first symptoms [29]. However, similar levels were observed in EA. The similarities between EA and early IBP could be explained by the lack of sensitivity to change of the SF-36 and also by the fact these two diseases are two inflammatory diseases with similar mechanisms. Furthermore, patients could have more expectations over time: the patient may change his internal standards, and/or conceptualization on the target [54]. Questionnaires reflect both true HRQoL and also how patients situate their level compared to what they would consider as ‘the best possible state’. This is sometimes called the ‘response shift’ [55]. Finally, personality traits may explain an important part of HRQoL, which would contribute to the stability of HRQoL assessment when ASDAS-CRP and DAS28-ESR improve [56, 57]. All these factors may explain the lack of substantial improvements in HRQoL. It is interesting to note the discordance between changes in HRQoL and in disease activity assessed by ASDAS-CRP and DAS28-ESR. Indeed, there is a good agreement in changes of these measures (Table 1). However, the improvement is more important clinically for disease activity than changes of HRQoL. Indeed, in EA, at baseline mean DAS28-ESR was 5.1 (1.3) (high disease activity) and at the last time-point DAS28-ESR was 2.7 (1.3) (moderate disease activity). In early IBP, ASDAS-CRP at baseline was 2.6 (0.9) (high disease activity) and at the final time-point 2.0 (0.9) (moderate disease activity). This suggests the complexity of HRQoL. This may reflect the multifactorial aspects of HRQoL and the link between physical and mental aspects of HRQoL, which varied similarly in the present study. This dissociation could also be explained by a part of circularity in the ASDAS-CRP and DAS28-ESR, which assessed not only objective aspects but also patient perception of disease activity. Moreover, only baseline PCS explained MCS in EA and only PCS and MCS at baseline in early IBP explained MCS over 5 to 8 years. In both diseases, for PCS, ASDAS-CRP/DAS28-ESR was found to be associated, as well as baseline HRQoL. However, the link between ASDAS-CRP/DAS28-ESR and HRQoL was incomplete. Indeed, ASDAS-CRP/DAS28-ESR explained only PCS (and only partly in particular in early IBP), not MCS over time. This indicates a link between physical aspects of HRQoL and disease activity, whereas mental aspects appeared independent from disease activity [54]. Mental HRQoL is a complex notion, probably encompassing personality traits, psychological distress and personal and social factors which may be only weakly linked to axSpA and RA [56]. Overall, baseline HRQoL was strongly associated to 5 to 8 years’ HRQoL, and indeed, it was the only predictor for MCS. This confirms previous studies where baseline levels strongly explained ulterior outcomes [42].

The present study has strengths and limitations. The two cohorts include patients with early rheumatic diseases: ESPOIR patients with definite synovitis for less than 6 months, of whom 81.4% satisfied the RA classification criteria, and DESIR patients with IBP, of whom 62.2% satisfied the ASAS classification criteria for axSpA [36, 37]. This is both a strength and a weakness. This reflects patients seen by rheumatologists at disease inception. Furthermore, analyses were checked on the subgroups of patients satisfying the axSpA and RA classification criteria, and results were similar. Socio-demographic factors associated with HRQoL were analysed; unfortunately, factors such as psychological distress and personality traits were not collected [56, 57]. There were missing data over the 5 to 8 years of follow-up. However, the size of the cohorts allowed full analyses, and for the regressions, linear-mixed models were applied to manage the missing data even if the bias of non-random missing data were persisted. In our study, HRQoL was assessed mainly using the SF36. SF-36 is the most widely used generic HRQoL questionnaire and allowed to compare the changes over time in two different populations, EA and early IBP [58]. However, other scores exist which are more specific to axSpA and RA [14, 40, 43, 59, 60]. Furthermore to assess correctly the evolution of HRQoL, group-level data were compared and trajectory-based mapping was performed [39, 40].

## Conclusions

HRQoL is announced as one of the objectives of inflammatory disease management. The present results indicate disjunctions between HRQoL and the immediate target of treatments, which is disease activity [61]. In the light of this, it is interesting to reflect on what falls within the remit of the rheumatologist and the health care team when addressing HRQoL. We argue the multifactorial nature of HRQoL may not make it the main relevant target, for example, in a treat-to-target approach [61].

In summary, in the era of personalized medicine and tailored treatment strategies, the identification of HRQoL evolution is important to improve patients’ management. The drivers of HRQoL in inflammatory rheumatic disorders and specifically the links with disease activity should be further explored.

## Additional files

Additional file 1: **Table S1.** Change of MCS and PCS SF-36 and disease activity over time. (DOCX 55 kb)

Additional file 2: **Table S2.** Characteristics of patients in each trajectory of 566 patients in early arthritis and 459 patients in early inflammatory back pain patients recruited to observational cohorts. (DOCX 54 kb)

Additional file 3: **Table S3.** Change of the SF-36 subdomains over 8 years in early arthritis and over 5 years in early inflammatory back pain IBP. (DOCX 55 kb)

## Abbreviations

ACR/EULAR: American College of Rheumatology/European League Against Rheumatism
ASAS: Assessment in Spondyloarthritis International Society
ASDAS-CRP: Ankylosing Spondylitis Disease Activity Score—C reactive protein
axSpA: Axial spondyloarthritis
DAS28-ESR: Disease Activity Score 28—erythrocyte sedimentation rate
DESIR: Devenir des Spondylarthropathies Indifférenciées Récentes
DMARDs: Conventional disease-modifying antirheumatic drugs
EA: Early arthritis
ESPOIR: Etude et Suivi des Polyarthrites Indifférenciées Récentes
HAQ: Health-Assessment Questionnaire
HRQoL: Health-related quality of life
IBP: Inflammatory back pain
MCS: Mental composite score
PCS: Physical composite score
RA: Rheumatoid arthritis
SF-36: 36-Item Short Form Survey
TNFi: Tumor necrosis factor inhibitors
US norms: United States norms
