# Supplementary material for: Similar alteration for mental and physical aspects in health-related quality of life over 5 to 8 years in 1347 patients with early arthritis and early inflammatory back pain
Source: Arthritis Res Ther. 2019 Feb 19;21:63. doi: 10.1186/s13075-019-1841-y (PMC6381682; doi:10.1186/s13075-019-1841-y)
Supplement: Supplementary file 1 — Table S1. Change of MCS and PCS SF-36 and disease activity over time. (DOCX 55 kb) [file 13075_2019_1841_MOESM1_ESM.docx]

Additional file 1: Table S1: Change of MCS and PCS SF-36 and disease activity over time

|  | **MCS** | **PCS** | **Number of patients** | **DAS 28-ESR** |
| --- | --- | --- | --- | --- |
|  | **EA** | | | |
| Time-point | Mean (sd) | Mean (sd) | N total (NA) | Mean (sd) |
| Baseline | 39.8 (10.9) | 38.5 (8.5) | 696 (5) | 5.1 (1.3) |
| Month 6 | 44.3 (11.0) | 44.3 (8.9) | 686 (15) | 3.4 (1.4) |
| Month 12 | 44.6 (11.4) | 44.8 (8.8) | 680 (21) | 3.2 (1.3) |
| Month 18 | 45.3 (11.1) | 45.4 (8.9) | 676 (25) | 3.0 (1.3) |
| Month 24 | 45.7 (11.2) | 45.1 (9.3) | 676 (25) | 2.9 (1.4) |
| Month 36 | 45.7 (11.1) | 44.9 (9.3) | 607 (94) | 2.9 (1.4) |
| Month 48 | 45.9 (11.0) | 44.7 (9.4) | 594 (107) | 2.8 (1.3) |
| Month 60 | 45.9 (11.0) | 45.1 (9.4) | 651 (140) | 2.8 (1.4) |
| Month 74 | 46.4 (10.5) | 45.0 (9.4) | 535 (166) | 2.8 (1.4) |
| Month 84 | 46.6 (10.1) | 45.3 (9.5) | 519 (182) | 2.7 (1.4) |
| Month 96 | 46.9 (10.2) | 44.9 (9.2) | 508 (193) | 2.7 (1.3) |
|  | **Early IBP** | | | |
|  | **MCS** | **PCS** |  | **ASDAS-CRP** |
| Time-point | Mean (sd) | Mean (sd) | N total (NA) | Mean (sd) |
| Baseline | 40.4 (11.2) | 40.2 (9.1) | 638 (8) | 2.6 (0.9) |
| Month 6 | 43.3 (11.5) | 42.5 (9.2) | 616 (30) | 2.2 (0.9) |
| Month 12 | 43.2 (11.4) | 42.4 (9.4) | 602 (44) | 2.2 (0.9) |
| Month 18 | 43.5 (11.3) | 42.9 (9.4) | 546 (100) | 2.1 (0.9) |
| Month 24 | 44.1 (11.2) | 42.5 (9.4) | 563 (83) | 2.2 (0.9) |
| Month 36 | 43.9 (11.4) | 42.5 (9.6) | 522 (124) | 2.1 (0.9) |
| Month 48 | 44.7 (10.5) | 43.2 (9.4) | 480 (166) | 2.1 (0.9) |
| Month 60 | 44.9 (11.1) | 43.1 (9.7) | 466 (180) | 2.0 (0.9) |

IBP: inflammatory back pain; EA: early arthritis; NA: not available
