# Supplementary material for: Similar alteration for mental and physical aspects in health-related quality of life over 5 to 8 years in 1347 patients with early arthritis and early inflammatory back pain
Source: Arthritis Res Ther. 2019 Feb 19;21:63. doi: 10.1186/s13075-019-1841-y (PMC6381682; doi:10.1186/s13075-019-1841-y)
Supplement: Supplementary file 2 — Table S2. Characteristics of patients in each trajectory of 566 patients in early arthritis and 459 patients in early inflammatory back pain patients recruited to observational cohorts. (DOCX 54 kb) [file 13075_2019_1841_MOESM2_ESM.docx]

Additional file 2: Table S2: Characteristics of patients in each trajectory of 566 patients in early arthritis and 459 patients in early inflammatory back pain patients recruited to observational cohorts

| **Characteristics at baseline** | EA population  Trajectory A  N = 344 | EA population  Trajectory B  N = 222 | P value | IBP population  Trajectory A  N = 268 | IBP population  Trajectory B  N = 191 | P value |
| --- | --- | --- | --- | --- | --- | --- |
|  | **MCS** | | | | | |
|  | N=344 | N=222 |  | N=268 | N=191 |  |
| Age (years), mean (SD) | 48.4 (11.9) | 50.5 (10.7) | 0.03 | 33.6 (8.8) | 35.3 (8.3) | 0.03 |
| Sex, females, N (%) | 251 (73.0) | 187 (84.2) | 0.002 | 142 (53.0) | 114 (59.7) | 0.13 |
| Studies above high school, N (%) | 129 (37.5) | 47 (21.2) | <0.001 | 186 (69.7) | 96 (50.3) | <0.001 |
| Smoking status, yes, N (%) | 167 (48.5) | 99 (44.6) | 0.36 | 77 (28.8) | 73 (38.6) | 0.02 |
| HAQ, mean (SD) | 0.84 (0.65) | 1.16 (0.68) | <0.001 | 0.35 (0.48) | 0.80 (0.45) | <0.001 |
| DAS28-ESR / ASDAS-CRP, mean (SD) | 4.95 (1.31) | 5.31 (1.26) | 0.001 | 2.44 (0.95) | 2.95 (0.96) | <0.001 |
| SF36-PCS, mean (SD) | 39.5 (8.7) | 36.9 (8.0) | <0.001 | 41.9 (9.5) | 38.6 (7.7) | <0.001 |
| SF36-MCS, mean (SD) | 44.2 (10.2) | 33.7 (8.8) | <0.001 | 45.5 (9.4) | 33.3 (8.7) | <0.001 |
|  |  | **PCS** | | | |  |
|  | N=324 | N=242 |  | N=249 | N=210 |  |
| Age (years), mean (SD) | 48.2 (12.3) | 50.6 (10.3) | 0.02 | 33.4 (8.7) | 35.4 (8.4) | 0.01 |
| Sex, females, N (%) | 253 (78.1) | 185 (77.3) | 0.64 | 112 (45.0) | 144 (68.6) | <0.001 |
| Studies above high school, N (%) | 129 (39.8) | 47 (19.4) | <0.001 | 171 (68.7) | 111 (52.9) | <0.001 |
| Smoking status, yes, N (%) | 146 (45.1) | 120 (49.6) | 0.29 | 74 (29.7) | 76 (36.2) | 0.15 |
| HAQ, mean (SD) | 0.75 (0.60) | 1.25 (0.68) | <0.001 | 0.46 (0.38) | 0.68 (0.47) | <0.001 |
| DAS28-ESR / ASDAS-CRP, mean (SD) | 4.81 (1.35) | 5.46 (1.15) | <0.001 | 2.37 (0.95) | 2.97 (0.92) | <0.001 |
| SF36-PCS, mean (SD) | 41.7 (7.8) | 34.1 (7.6) | <0.001 | 45.3 (7.6) | 35.0 (7.1) | <0.001 |
| SF36-MCS, mean (SD) | 42.1 (10.5) | 37.4 (10.9) | <0.001 | 42.9 (10.8) | 37.4 (10.3) | <0.001 |
