# Supplementary material for: Similar alteration for mental and physical aspects in health-related quality of life over 5 to 8 years in 1347 patients with early arthritis and early inflammatory back pain
Source: Arthritis Res Ther. 2019 Feb 19;21:63. doi: 10.1186/s13075-019-1841-y (PMC6381682; doi:10.1186/s13075-019-1841-y)
Supplement: Supplementary file 3 — Table S3. Change of the SF-36 subdomains over 8 years in early arthritis and over 5 years in early inflammatory back pain IBP. (DOCX 55 kb) [file 13075_2019_1841_MOESM3_ESM.docx]

Additional file 3: Table S3: Change of the SF-36 subdomains over 8 years in early arthritis and over 5 years in early inflammatory back pain IBP

|  | **PF** | **PR** | **BP** | **GH** | **VT** | **SF** | **ER** | **MH** |
| --- | --- | --- | --- | --- | --- | --- | --- | --- |
| **EA** | | | | | | | | |
| Baseline | 58.0 (26.2) | 32.1 (37.5) | 37.9 (20.4) | 56.2 (17.7) | 39.7 (19.3) | 60.0 (25.5) | 41.5 (42.1) | 53.0 (19.7) |
| Month 6 | 71.6 (24.2) | 60.9 (40.8) | 58.2 (21.8) | 57.7 (18.1) | 49.3 (19.8) | 72.5 (24.5) | 66.1 (41.0) | 60.0 (20.1) |
| Month 12 | 72.9 (24.4) | 62.8 (40.3) | 60.3 (24.06) | 57.7 (19.2) | 50.3 (20.7) | 74.6 (24.0) | 66.1 (41.4) | 61.1 (21.04) |
| Year 3 | 73.2 (25.7) | 65.6 (40.1) | 62.1 (24.0) | 57.2 (20.1) | 51.3 (21.7) | 76.3 (23.8) | 70.3 (39.9) | 62.8 (21.0) |
| Year 8 | 72.9 (26.3) | 68.2 (40.2) | 63.3 (24.7) | 58.2 (19.1) | 52.2 (19.7) | 78.1 (23.2) | 73.0 (40.0) | 64.9 (19.8) |
| Delta Baseline-Year 8 | 14.9 | 36.1 | 25.3 | 2.0 | 12.5 | 18.2 | 31.4 | 11.9 |
| **IBP** | | | | | | | | |
| M0 | 66.9 (24.3) | 46.1 (39.8) | 44.2 (21.0) | 47.7 (21.0) | 37.3 (19.7) | 58.9 (27.0) | 54.7 (41.6) | 56.2 (19.7) |
| Month 6 | 72.4 (23.3) | 57.5 (41.1) | 52.9 (23.3) | 50.2 (22.3) | 43.7 (20.6) | 66.9 (26.9) | 65.3 (41.0) | 60.6 (20.1) |
| Month 12 |  |  |  |  |  |  |  |  |
| Year 3 | 74.2 (23.2) | 57.9 (42.1) | 55.1 (24.3) | 50.3 (22.6) | 43.7 (21.0) | 69.7 (26.2) | 67.0 (41.5) | 61.5 (20.0) |
| Year 5 | 74.2 (23.5) | 62.5 (40.4) | 56.4 (23.7) | 49.4 (22.2) | 45.0 (21.7) | 72.8 (26.0) | 71.1 (39.7) | 62.2 (19.6) |
| Delta Baseline-Year 5 | 7.3 | 16.1 | 12.2 | 1.7 | 7.7 | 13.9 | 16.4 | 6.0 |

The 8 subdomains of SF36 are: PF: Physical Function; PR: Physical Role; BP: Bodily Pain; GH: General Health; VT: Vitality; SF: Social Function; ER: Emotional Role and MH: Mental Health; Results are mean (SD) The French population norms for SF-36 subdomains: Physical Function 86.5; Physical Role 80.6; Bodily Pain 74.3; General Health 69.0; Vitality 61.4; Social Function 80.7; Emotional Role 80.9 and Mental Health 67.8.
